# Ionising Radiation Immediately Impairs Synaptic Plasticity-Associated Cytoskeletal Signalling Pathways in HT22 Cells and in Mouse Brain: An *In Vitro/In Vivo* Comparison Study

**DOI:** 10.1371/journal.pone.0110464

**Published:** 2014-10-20

**Authors:** Stefan J. Kempf, Sonja Buratovic, Christine von Toerne, Simone Moertl, Bo Stenerlöw, Stefanie M. Hauck, Michael J. Atkinson, Per Eriksson, Soile Tapio

**Affiliations:** 1 Institute of Radiation Biology, Helmholtz Zentrum München, German Research Center for Environmental Health GmbH, Neuherberg, Germany; 2 Department of Environmental Toxicology, Uppsala University, Uppsala, Sweden; 3 Research Unit Protein Science, Helmholtz Zentrum München, German Research Center for Environmental Health GmbH, Neuherberg, Germany; 4 Division of Biomedical Radiation Sciences, Rudbeck Laboratory, Uppsala University, Uppsala, Sweden; 5 Chair of Radiation Biology, Technical University Munich, Munich, Germany; University of South Alabama, United States of America

## Abstract

Patients suffering from brain malignancies are treated with high-dose ionising radiation. However, this may lead to severe learning and memory impairment. Preventive treatments to minimise these side effects have not been possible due to the lack of knowledge of the involved signalling pathways and molecular targets. Mouse hippocampal neuronal HT22 cells were irradiated with acute gamma doses of 0.5 Gy, 1.0 Gy and 4.0 Gy. Changes in the cellular proteome were investigated by isotope-coded protein label technology and tandem mass spectrometry after 4 and 24 hours. To compare the findings with the *in vivo* response, male NMRI mice were irradiated on postnatal day 10 with a gamma dose of 1.0 Gy, followed by evaluation of the cellular proteome of hippocampus and cortex 24 hours post-irradiation. Analysis of the *in vitro* proteome showed that signalling pathways related to synaptic actin-remodelling were significantly affected at 1.0 Gy and 4.0 Gy but not at 0.5 Gy after 4 and 24 hours. We observed radiation-induced reduction of the miR-132 and Rac1 levels; miR-132 is known to regulate Rac1 activity by blocking the GTPase-activating protein p250GAP. In the irradiated hippocampus and cortex we observed alterations in the signalling pathways similar to those *in vitro*. The decreased expression of miR-132 and Rac1 was associated with an increase in hippocampal cofilin and phospho-cofilin. The Rac1-Cofilin pathway is involved in the modulation of synaptic actin filament formation that is necessary for correct spine and synapse morphology to enable processes of learning and memory. We suggest that acute radiation exposure leads to rapid dendritic spine and synapse morphology alterations via aberrant cytoskeletal signalling and processing and that this is associated with the immediate neurocognitive side effects observed in patients treated with ionising radiation.

## Introduction

Ionising radiation is frequently used during treatment of central nervous system (CNS) malignancies. Normally, the patient is exposed to a total radiation dose of 20–50 Gy that is given in fractions of 2–4 Gy to reduce the side-effects. Still, immediate detrimental decline in cognition and visual memory are widely observed [1], [2]. Epidemiological studies indicate that even moderate radiation doses may lead to acute and permanent deficits in learning and memory [3]–[5], in particular if the exposure occurred during childhood [6], [7].

Approximately 200,000 children worldwide were treated with X-rays for ringworm of the scalp (*Tinea capitis*), with head doses ranging from 0.7 to 1.7 Gy [8], [9]: Long-term side-effects on cognition were evaluated 10 to 29 years later, showing that psychiatric disorders were more often diagnosed in exposed children than in not exposed ones [10]. A follow-up study with 11,000 irradiated Israeli *Tinea capitis* children showed similar long-term effects after radiation exposure including lower examination scores, intelligence quotients, and a small increase in the frequency of mental retardation [4].

The cognitive damage in people exposed early in life may be a consequence of the immature state of the brain when ionising radiation was applied. On its way to adolescence, the brain undergoes various remodelling processes on molecular and structural levels called the brain growth spurt [11]. It includes fundamental neuronal architecture changes such as growth of axons and dendrites to enable the formation and deletion of synaptic contacts [12]. The brain is especially susceptible to damage if exposed to ionising radiation during this developmental period. It has been shown that toxic agents given to mice within the susceptibility window around postnatal day ten lead to disruption of adult brain function [13], [14]. Further, a synergistic effect between toxicants and ionising radiation given on postnatal day ten has been shown [15]. Interestingly, the brain growth spurt is species-dependent as in human beings it lasts until the age of three to four years whereas in rodents it corresponds to the second and fourth postnatal weeks [16].

Especially the hippocampus is a highly radiation-sensitive brain region involved in learning and memory consolidation. Irradiation may lead to changes in the neurogenic niche of the dentate gyrus of the hippocampus by depleting neural stem and progenitor cells [17]–[20]. Nevertheless, the low frequency of life-long newly generated neurons in this region may suggest that other brain regions and biological targets may also be of importance in the manifestation of long-lasting cognitive defects after radiation treatment. It has been shown recently that the mature neuronal networks of the hippocampus are highly-radiation sensitive [21]. Thus, ionising radiation may have adverse effects on the effective neurotransmission by altering the synaptic plasticity of the brain. Synaptic plasticity is a dynamic process involving rapid cytoskeletal organisation on the dendrite and spine morphology to modulate signal transmission. Defects in synaptic plasticity and dendrite or spine morphology have been observed in cognitive diseases such as Alzheimer's [22], Rett syndrome [23] and Down's syndrome [24], emphasising not only the role of the hippocampus but also that of the cortex in this process.

The aim of this study was (i) to determine the role of synaptic plasticity-associated cytoskeletal signalling pathways in the acute radiation response *in vitro* and *in vivo* and (ii) to compare these alterations. We show here that dendritic spine morphology-associated proteins and signalling pathways such as the Rac1-Cofilin pathway were rapidly altered after *in vitro* exposure to a dose of 1.0 Gy in primary immortalised neurons of the mouse hippocampal cells (HT22). Similar alterations were confirmed in the hippocampus and cortex of NMRI mice irradiated on postnatal day ten that represents a developmental stage within the brain growth spurt in mice.

## Materials and Methods

### Ethics statement, irradiation of animals and tissue collection

Experiments were carried out in accordance with the European Communities Council Directive of 24 November 1986 (86/609/EEC), after approval from the local ethical committees (Uppsala University and the Agricultural Research Council) and by the Swedish Committee for Ethical Experiments on Laboratory Animals. All animal experiments were performed under trained personnel, and all efforts were made to minimise animal suffering.

Male NMRI mice were total body irradiated on postnatal day 10 (PND 10) with a single exposure to gamma irradiation (^137^Cs, 0.20 Gy/min) at doses of 0 (sham-irradiated control) and 1.0 Gy (Rudbeck Laboratory, Uppsala University). Dose verification was done with an ionisation chamber (Markus chamber type 23343, PTW-Freiburg) and was homogeneous within ±3% over the 10 cm dish area where mice were positioned during irradiation procedure. Neonates from each litter were irradiated together.

Animals were sacrificed via cervical dislocation. Brains were excised and transferred to ice-cold PBS, rinsed carefully, and dissected under stereomicroscopic inspection under cold conditions. Hippocampi and cortices without meninges from each hemisphere were separately sampled, gently rinsed in ice-cold PBS and snap-frozen in liquid nitrogen. Samples were stored at −80°C until isolation of protein and RNA.

### Irradiation and harvesting of cells

HT22 cells (immortalised primary neurons from the mouse hippocampus) were kindly provided from J. Lewerenz (Department of Neurology, University Hospital Hamburg-Eppendorf, Hamburg, Germany) [25]. The cells were grown in high glucose DMEM media (PAA Laboratories, E15-840) supplemented with 10% foetal bovine serum (PAA Laboratories, A15-101) without antibiotics in T75 tissue flask at 37°C with 5% C0_2_ in air. They were irradiated in the exponential growth phase with doses of 0 Gy (sham), 0.5 Gy, 1.0 Gy or 4.0 Gy of γ-rays (^137^Cs, 0.48 Gy/min) (HWM-D 2000, Waelischmiller Engineering, Germany). For each dose group and time point, four independent flasks were seeded and irradiated. At four and 24 hours post-irradiation cells were rinsed with ice-cold PBS and enzymatically detached with accutase (Invitrogen). After blocking of the accutase reaction with media containing 10% foetal bovine serum and splitting of cell volume in two equal parts for total RNA and protein isolation of each tissue flask, the cells were centrifuged and washed once with ice-cold PBS. This centrifugation and washing step was repeated, followed by cell pelleting via centrifugation. Pelleted cells were frozen at −80°C until total protein and RNA content were isolated.

### Isolation of total protein and RNA

#### a) Isolation of total protein

HT22 cell pellets or individual frozen hippocampi and cortices were homogenised with 6 M guanidine hydrochloride (SERVA Electrophoresis GmbH, Germany) on ice using a manual plastic mortar. Homogenates were briefly vortexed, sonicated, and cleared by centrifugation (20,000×g, 1 hour, 4°C). The supernatants were collected and stored at −20°C before further use. Total protein content was determined using Bradford assay (Thermo Fisher) following the manufacturer's instructions.

#### b) Total RNA isolation

Total RNA from HT22 cell pellets or individual frozen hippocampi and cortices was isolated and purified by mirVana™ Isolation Kit (Ambion) according to the manufacturer's instructions. Total RNA was eluted with nuclease-free water. The optical density (OD) ratio of 260/280 was measured using a Nanodrop spectrophotometer (PeqLab Biotechnology; Germany); it ranged between 1.9 and 2.1. Eluates were stored at −20°C until further analysis.

### Mass spectrometry-based proteome analysis

#### a) Isotope coded protein label (ICPL) analysis of proteins, 1D PAGE separation and in-gel digest

In total, four individual replicates of HT22 cells were used for proteomic analysis at each radiation dose and time point. Total protein lysates were labelled with ICPL reagents (SERVA Electrophoresis GmbH, Germany) according to the manufacturer's instructions. Briefly, individual protein lysates (20 µg in 20 µl of 6 M guanidine hydrochloride from each biological sample) were reduced, alkylated and labelled with the respective ICPL-reagent as follows: control with ICPL-0, 0.5 Gy sample with ICPL-4, 1.0 Gy sample with ICPL-6 and 4.0 Gy sample with ICPL-10. All labelled samples representing each radiation dose at one time point (4 and 24 hours) were combined and overnight precipitated with 80% acetone at −20°C to purify the labelled protein content.

Biological replicates from the *in vivo* mouse study included animals from at least three different litters. Four biological replicates from hippocampus and five from cortex were used for both control and irradiated groups. The samples were labelled with ICPL reagents as follows: control with ICPL-0 and 1.0 Gy sample with ICPL-6. These labelled samples were further treated as described for the HT22 cells.

Protein precipitates were separated by 12% SDS-polyacrylamide gel electrophoresis followed by Coomassie Blue staining. Gel lanes were cut into at least four equal slices, destained, and trypsinised overnight as described recently [26]. Peptides were extracted and acidified with 1% formic acid followed by analysis via mass spectrometry.

#### b) LC-MS/MS analysis

LC-MS/MS analysis was performed as described previously on a LTQ-Orbitrap XL (Thermo Fisher) [27]. Briefly, pre-fractionated samples were automatically injected and loaded onto the trap column and after 5 min, peptides were eluted and separated on the analytical column by reversed phase chromatography operated on a nano-HPLC (Ultimate 3000, Dionex) with a nonlinear 170 min gradient using 35% acetonitrile in 0.1% formic acid in water (A) and 0.1% formic acid in 98% acetonitrile (B) at a flow rate of 300 nl/min. The gradient settings were: 5–140 min: 14.5–90% A, 140–145 min: 90% A −95% B, 145–150 min: 95% B followed by equilibration for 15 min to starting conditions. From the MS pre-scan, the 10 most abundant peptide ions were selected for fragmentation in the linear ion trap if they exceeded an intensity of at least 200 counts and were at least doubly charged. During fragment analysis, a high-resolution (60,000 full-width half maximum) MS spectrum was acquired in the Orbitrap with a mass range from 200 to 1500 Da.

#### c) Identification and quantification of proteins

MS-MS spectra were searched against the ENSEMBL mouse database (Version: 2.4, 56416 sequences) via MASCOT (version 2.3.02; Matrix Science) with a mass tolerance of 10 ppm for peptide precursors and 0.6 Da for MS-MS peptide fragments, including not more than one missed cleavage. Fixed modifications included carbamidomethylation of cysteine and ICPL-0, ICPL-4, ICPL-6 and ICPL-10 for lysine. Proteins were identified and quantified based on the ICPL pairs using the Proteome Discoverer software (Version 1.3– Thermo Fisher). To ensure that only high-confident identified peptides were used for protein quantification, the MASCOT percolator algorithm was applied [28]. The percolator is an algorithm that improves the discrimination between correct and incorrect spectrum identifications and gives a q value sising the statistical confidence assigned to each peptide-spectra-match [29]. The q value was set to 0.01 representing strict peptide ranking. Only the best ranked peptides were used. Such peptides were filtered against Decoy database resulting into a false discovery rate (FDR) of each LC-MS-run; the significance threshold was set to 0.01 to ensure that only highly confident peptide identifications were used for protein quantification. Proteins from each LC-MS-run were normalised against the median of all quantifiable proteins. Proteins were considered significantly deregulated if they fulfilled the following criteria: (i) identification by at least two unique peptides in n-1 mass-spectrometry runs (n: number of biological replicates), (ii) quantification with an ICPL-variability of ≤30% and (iii) a fold-change of ≥1.3 or ≤ −1.3. The threshold of ±1.3 is based on our average experimental technical variance of the multiple analysis of hippocampal and cortical technical replicates (13.8%).

### Data deposition of proteomics experiments

The raw-files of the obtained MS-MS spectra can be found under http://storedb.org/project_details.php?projectid=38 with the ProjectID 38.

### Bioinformatics analysis

Deregulated proteins were assigned to functional classes using PANTHER classification system software (http://www.pantherdb.org) and the general annotation from UniProt (http://uniprot.org). To identify radiation-affected signalling pathways, a signalling pathway analysis was performed with all deregulated proteins for each dose group using INGENUITY Pathway Analysis (IPA) (http://www.ingenuity.com) applying databases of experimental and predictive origin.

### Quantification of Rac1, cofilin and phospho-cofilin expression levels via immunoblotting

Protein extracts of cells and brain tissues (15 µg) were separated on 12% SDS polyacrylamide gels and transferred to nitrocellulose membranes (GE Healthcare) via BIO-RAD Criterion™ Blotter system at 100 V for 2 h. Membranes were blocked with Roti^R^-Block solution (Roth), washed and incubated overnight at 4°C with primary antibody dilutions as recommended by the manufacturer (GAPDH – sc-47724 [murine monoclonal IgG1 raised against recombinant GAPDH of human origin; Santa Cruz], Rac1– ab33186 [murine monoclonal IgG2b raised against full-length recombinant Rac1 of human origin; Abcam], cofilin –3312 [rabbit polyclonal antibody produced by immunising rabbits with a synthetic peptide corresponding to residues surrounding Ser3 of human cofilin origin; Cell Signalling], p-Cofilin (Ser3) –3311 [rabbit polyclonal antibody by immunising animals with a synthetic phospho-peptide corresponding to residues surrounding Ser3 of human cofilin; Cell Signalling]). After a washing step, blots were incubated with appropriate horseradish peroxidase-conjugated secondary antibody in 8% milk for 1 h at room temperature and developed using ECL system (GE Healthcare) using standard protocol from the manufacturer. GAPDH was not significantly deregulated based on the global proteomics results in any sample and was therefore used as a loading control. Immunoblots were quantified with TotalLab TL100 software (www.totallab.com) using software-suggested background correction. Three or four biological replicates were used for statistical analysis (unpaired Student's t-test) with a significance threshold of 0.05.

### Quantification of microRNA miR-132 via quantitative PCR

RNA isolates of cells and brain tissues (10 ng) were used to quantify microRNA miR-132 expression levels using the TaqMan Single MicroRNA Assay (Applied Biosystems) according to the manufacturer's protocol. Steps included a reverse transcription and real-time PCR (StepOnePlus) via Taqman-primers (mmu-miR-132 (ID000457), snoRNA135 (ID001239) – Life Technologies). Expression levels of miRNA were calculated based on the 2^−ΔΔCt^ method with normalisation against endogenous snoRNA135 [30]. Changes were considered significant if they reached a p-value of ≤0.05 (unpaired Student's t-test, n = 4 [*in vitro*] and n = 3 [*in vivo*] per dose group and time point).

## Results

### Acute effects of ionising radiation on synaptic plasticity-associated cytoskeletal signalling pathways *in vitro*

**Figure 1:**
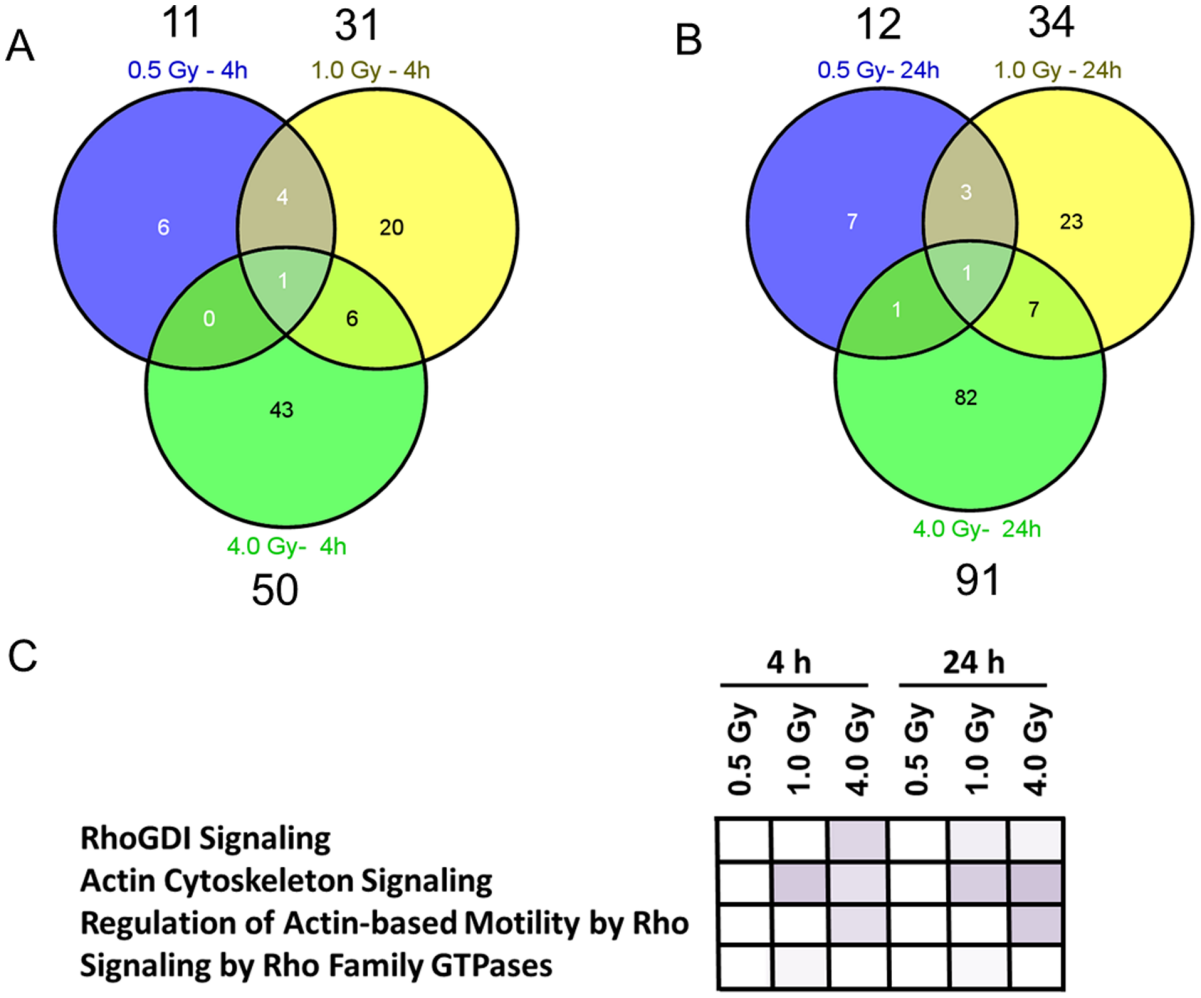
Mass spectrometry-based proteomics of *in vitro* irradiated HT22 cells. Venn diagrams showing the number of all and shared deregulated proteins from HT22 cells exposed to 0.5 Gy, 1.0 Gy and 4.0 Gy 4 hours (A) and 24 hours (B) post-irradiation using global proteomics approach; n = 4. The number above each dose shows the total number of deregulated proteins at this dose. Altered cytoskeletal signalling pathways at all doses using the Ingenuity Pathway Analysis (IPA) software are shown (C). Higher colour intensity represents higher significance (p-value) of the pathway. All coloured boxes have a p-value of ≤0.05; white boxes have a p-value of ≥0.05 and are not significantly altered. p-values at 4 hours: RhoGDI signalling (0.5 Gy: 0.3, 1.0 Gy: 0.289, 4.0 Gy: 0.0155), actin cytoskeleton signalling (0.5 Gy: 0.4, 1.0 Gy: 0.00819, 4.0 Gy: 0.0267), regulation of actin-based motility by Rho (0.5 Gy: 0.45, 1.0 Gy: 0.147, 4.0 Gy: 0.025), signalling by Rho Family GTPases (0.5 Gy: 0.4, 1.0 Gy: 0.049, 4.0 Gy: 0.43). p-values at 24 hours: RhoGDI signalling (0.5 Gy: 0.3, 1.0 Gy: 0.043, 4.0 Gy: 0.044), actin cytoskeleton signalling (0.5 Gy: 0.49, 1.0 Gy: 0.0106, 4.0 Gy: 0.00608), regulation of actin-based motility by Rho (0.5 Gy: 0.51, 1.0 Gy: 0.16, 4.0 Gy: 0.00971), signalling by Rho Family GTPases (0.5 Gy: 0.53, 1.0 Gy: 0.049, 4.0 Gy: 0.13).

We used HT22 cells as an *in vitro* model to detect radiation-induced alterations in synaptic plasticity-associated cytoskeletal signalling pathways. HT22 cells were irradiated with doses of 0.5 Gy, 1.0 Gy and 4.0 Gy, followed by a global quantitative proteome analysis 4 and 24 hours post-irradiation. The protein quantification showed a dose-dependency in the number of significantly deregulated proteins (4 h/24 h: 0.5 Gy −1/12, 1.0 Gy –31/34, 4.0 Gy –50/91) (Figure 1 A and B). Table S1 in File S2 shows the complete list of deregulated proteins. The Venn diagrams in Figure S1 A – C in File S1 show the overlapping proteins between the two time points (4 and 24 hours) at 0.5 Gy, 1.0 Gy and 4.0 Gy. Importantly, all overlapping proteins showed the same direction of deregulation at both time points (Figure S1 D – F in File S1).

Bioinformatics analysis of signalling pathways using the Ingenuity Pathway Analysis (IPA) software showed that synaptic-plasticity associated cytoskeletal remodelling pathways were affected by radiation exposure in HT22 cells. RhoGDI signalling (4 h: 4.0 Gy; 24 h: 1.0 Gy, 4.0 Gy – p<0.05), actin cytoskeleton signalling (4 h: 1.0 Gy, 4.0 Gy; 24 h: 1.0 Gy, 4.0 Gy – p<0.05), regulation of actin-based motility by Rho (4 h: 4.0 Gy; 24 h: 4.0 Gy – p<0.05) and signalling by Rho family GTPases (4 h: 1.0 Gy; 24 h: 1.0 Gy – p<0.05) were the most important pathways affected (Figure 1 C). Importantly, these pathways were not significantly altered at 0.5 Gy for any time point (Figure 1 C). The shared deregulated proteins from each pathway are shown in Figure S2 in File S1 consisting of the Rho family GTPase Rac, the kinases PAK and LIMK and the actin cytoskeleton-remodelling cofilin. All these proteins are involved in axonal maturation, spine- and synapse formation, maturation and -morphology via regulation of actin polymerisation (Rac1-Cofilin pathway) [31]–[33].

### Ionising radiation impairs the RhoGTPase Rac1 *in vitro*

**Figure 2:**
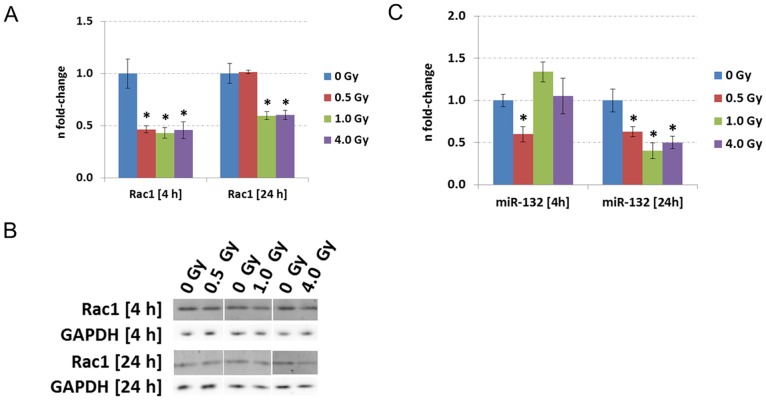
Immunoblotting and miRNA quantification of using HT22 cells. Data from immunoblotting (A–B) and miRNA quantification (C) associated to the Rac1-Cofilin pathway in HT22 cells irradiated with 0 Gy, 0.5 Gy, 1.0 Gy and 4.0 Gy 4 hours and 24 hours post-irradiation. The columns represent the fold-changes with standard errors of the mean (SEM); n = 3 for immunoblotting, n = 4 for miRNA quantification. The visualisation of protein bands shows the representative change from the biological replicates. *p<0.05; **p<0.01; ***p<0.001 (unpaired Student's t-test). Normalisation was performed against endogenous GAPDH and endogenous snoRNA135 for immunoblotting and miRNA quantification, respectively.

To validate the radiation-induced change in the Rac1-Cofilin pathway in HT22 cells, the Rho family GTPase Rac1 as the main upstream modulator of this pathway was quantified via immunoblotting. The analysis demonstrated that the expression levels of the Rac1 protein were significantly down-regulated at 1.0 Gy and 4.0 Gy at both time points (Figure 2 A and B). The Rac1 protein levels at 0.5 Gy were significantly decreased at 4 hours but returned to control levels after 24 hours post-irradiation (Figure 2 A and B). These data are in good agreement with the changes in Rac1 expression levels obtained by global proteomics approach at 24 hours post-irradiation (1.0 Gy: −1.43±20.3%; 4.0 Gy: −1.40±15.9%) (Table S1 in File S2).

As miR-132 is involved in the regulation of the Rac1-Cofilin pathway via blocking of the GTP hydrolysis protein p250GAP [31], the levels of this microRNA were quantified. The miR-132 levels were significantly decreased at 0.5 Gy but not at higher doses 4 hours post-irradiation whereas 24 hours post-irradiation miR-132 levels were still significantly decreased at 0.5 Gy and were also decreased at the higher doses (Figure 2 C). Importantly, the changes observed for miR-132 expression originated from the alterations in the expression of miR-132 and not in the expression of endogenous standard snoRNA135 (Figure S5 in File S1). Figures S5 E and S5 F in File S1 show the variation of the n-fold changes of snoRNA135 used for miRNA normalisation after 4 hours and 24 hours post-irradiation and the ΔCt values between miR-132 and snoRNA135 in irradiated HT22 cells, respectively. Only small variances in the expression of snoRNA135 were detectable whereas the ΔCt values were more affected meaning that miRNA changes originated from radiation-induced differences in miR-132 expression profile. The error bars were in all conditions comparable.

### Acute effects of ionising radiation on synaptic plasticity-associated cytoskeleton signalling pathways *in vivo*

To examine possible radiation-induced effects on spine- and synapse formation, maturation and morphology, we irradiated male NMRI mice on postnatal day ten – a developmental stage of maximal cytoskeleton remodelling in dendritic spines and synapses within the brain growth spurt period. We irradiated neonates using a dose of 1.0 Gy and performed experiments 24 hours post-irradiation as this dose was the lowest showing persistent effect on the Rac1 expression and signalling pathway alterations in the irradiated HT22 cells (Figure 1 and 2). The analysis was performed using both hippocampus and cortex as these two brain regions are involved in learning and memory formation [34], [35].

**Figure 3:**
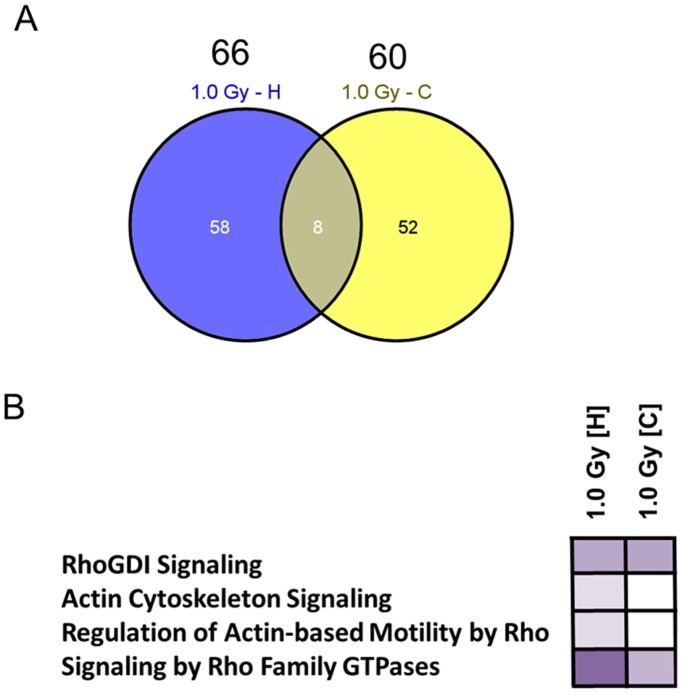
Mass spectrometry-based proteomics of the hippocampus and cortex of irradiated NMRI mice. Venn diagram of all deregulated and shared hippocampal [H] and cortical [C] proteins from global proteomics analysis using doses of 0 Gy and 1.0 Gy 24 hours post-irradiation (A). Hippocampus: n = 4 and cortex: n = 5. The number above each dose shows the total number of deregulated proteins at this dose. Associated cytoskeletal signalling pathways of all deregulated proteins using the Ingenuity Pathway Analysis (IPA) software in hippocampus [H] and cortex [C] are shown in (B). Higher colour intensity represents higher significance (p-value) whereas all coloured boxes have a p-value of ≤0.05; white boxes have a p-value of ≥0.05 and are not significantly altered. p-values: RhoGDI signalling (hippocampus: 0.000535, cortex: 0.000459), actin cytoskeleton signalling (hippocampus: 0.049, cortex: 0.189), regulation of actin-based motility by Rho (hippocampus: 0.0389, cortex: 0.261), signalling by Rho Family GTPases (hippocampus: 0.00000271, cortex: 0.00166).

The protein quantification via global proteomics showed that 66 and 60 proteins were deregulated in the hippocampus and cortex 24 hours after exposure to 1.0 Gy, respectively (Figure 3 A). Eight proteins were found to be shared and up-regulated in these two brain regions (Figure S3 in File S1). Table S2 in File S2 shows the complete list of the *in vivo* deregulated proteins.

Signalling pathway analysis using the IPA software tool demonstrated that, similar to the *in vitro* cellular study, synaptic-plasticity associated cytoskeletal pathways were affected *in vivo* by radiation exposure: RhoGDI signalling (hippocampus and cortex – p<0.05), actin cytoskeleton signalling (hippocampus – p<0.05), regulation of actin-based motility by Rho (hippocampus – p<0.05) and signalling by Rho family GTPases (hippocampus and cortex – p<0.05) (Figure 3 B).

### Ionising radiation impairs the RhoGTPase Rac1 and its downstream target cofilin *in vivo*

**Figure 4:**
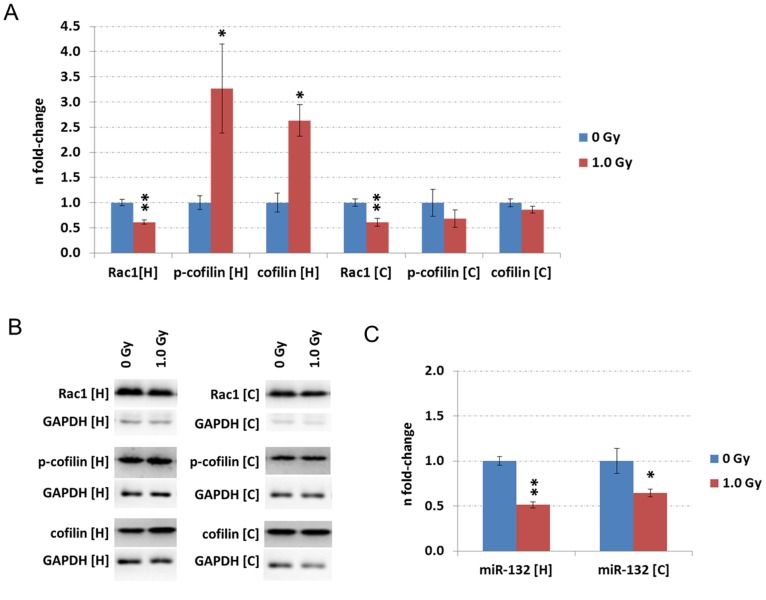
Immunoblotting and miRNA quantification of the *in vivo* data. Data from immunoblotting (A–B) and miRNA quantification (C) associated to the Rac1-Cofilin pathway in hippocampus [H] and cortex [C] from NMRI mice exposed on postnatal day 10 with doses of 0 Gy and 1.0 Gy. The measurement was performed 24 hours post-irradiation. The columns represent the fold-changes with standard errors of the mean (SEM); immunoblotting: n = 4 for Rac1 detection; n = 3 for p-cofilin and cofilin detection; n = 3 for miRNA quantification. The visualisation of protein bands shows the representative change from the biological replicates. *p<0.05; **p<0.01; ***p<0.001 (unpaired Student's t-test). Normalisation was performed against endogenous GAPDH and endogenous snoRNA135 for immunoblotting and miRNA quantification, respectively.

Quantification of Rac1 protein levels by immunoblotting confirmed a significant decrease in hippocampus and cortex at 1.0 Gy (Figure 4 A and B) to a similar extent as in the HT22 cells (Figure 2 A and B). In accordance with decreasing miR-132 levels in HT22 cells 24 hours post-irradiation with 1.0 Gy (Figure 2 C), we observed significantly decreased miR-132 levels in both hippocampus and cortex at 1.0 Gy 24 hours post-irradiation (Figure 4 C). HT22 data highlighted that the observed deregulation of miR-132 is due to miR-132 deregulation itself (Figure S5 E and S5 F in File S1). Similar results were obtained in the evaluation of hippocampal and cortical ΔCt values of miR-132 and snoRNA135 (Figure S5 A – S5 D in File S1).

To further evaluate possible downstream effects of Rac1 in the Rac1-Cofilin pathway, we quantified the expression of cofilin as well as phosphorylated cofilin which is the inactive form. Immunoblotting showed that both cofilin and phospho-cofilin levels were significantly increased in the hippocampus by irradiation with 1.0 Gy but there was no significant expression alteration in the cortex (Figure 4 A and B).

### Comparison of radiation-induced proteome changes *in vitro* and *in vivo*

**Figure 5:**
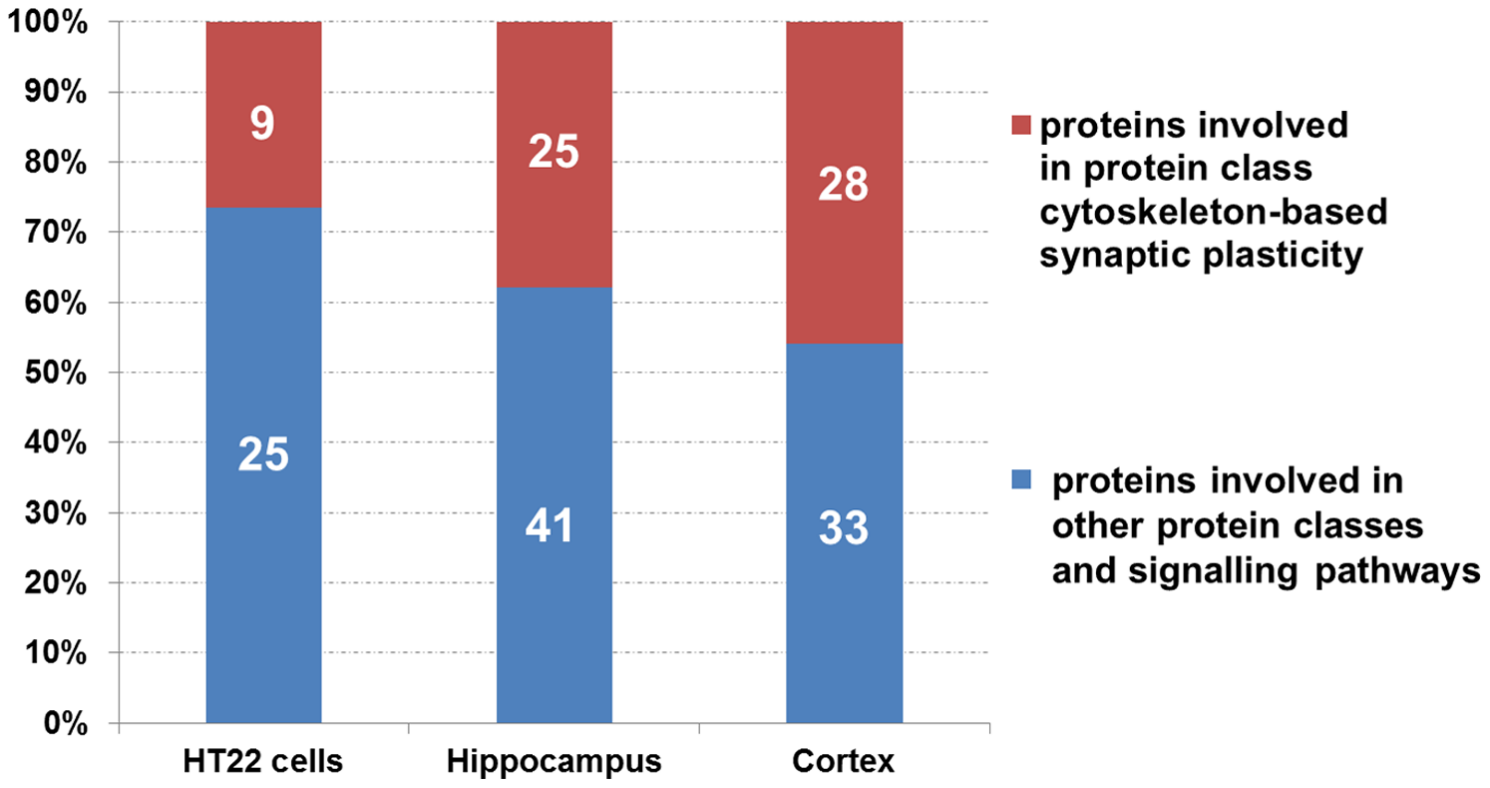
Mass spectrometry-based proteomics - comparison of the *in vitro* and *in vivo* data. The number of deregulated proteins from the *in vitro* and *in vivo* global proteomics analysis (1.0 Gy, 24 hours post-irradiation) belonging to the protein class “cytoskeleton-based synaptic plasticity” (Table S1 and S2) compared to all deregulated proteins in the respective cellular/organ system. The figure shows that 9 (26.5%), 25 (37.9%) and 28 (45.9%) proteins from all deregulated proteins of HT22 cells, hippocampus and cortex, respectively, can be grouped into this class. This class is based on the protein affiliations into sub-protein classes obtained from the PANTHER software (PANTHER protein class) as shown in Table S1 and S2 (PANTHER protein classes are highlighted in orange) involving cytoskeleton -associated processes, G-protein -associated processes and cell adhesion -associated processes.

Deregulated proteins from *in vitro* cellular experiments (24 hours, 1.0 Gy) and from murine hippocampus and cortex (24 hours, 1.0 Gy) were grouped according to protein affiliations by the PANTHER software tool (Table S1 and S2 in File S2). The analysis showed that a high degree of proteins were categorised into protein classes involved in (i) cytoskeleton-associated processes, (ii) G-protein-associated processes and (iii) cell adhesion-associated processes (Table S1 and S2 in File S2– PANTHER protein classes highlighted in orange). Proteins involved in these classes were then manually curated for the class “cytoskeleton-based synaptic plasticity” based on literature [36], [37] and the UniProt database. In total, 9 out of 34 proteins, 25 out of 66 proteins and 28 out of 61 proteins from all deregulated proteins in HT22 cells, hippocampus and cortex, respectively, were found to belong to this class of proteins (Figure 5). The percentage of synaptic plasticity proteins of all deregulated proteins is shown in Figure 5. Thus, we observed an increase in deregulated proteins associated to synaptic-cytoskeletal processes after acute radiation exposure in HT22 cells (26.5%) as well as hippocampus (37.9%) and cortex (45.9%) (Figure 5). Proteins involved in other protein classes and signalling pathways act as transcription−/translation-factors, general metabolic enzymes or transfers/carriers (Table S1 and S2 in File S2). It is important to note that only one protein (Gmps - GMP synthase) was overall shared and up-regulated (Figure S4 A and S4 B in File S1). This protein is involved in the *de novo* synthesis of guanine nucleotides to provide GTP.

Overall, these results suggest that synaptic cytoskeleton-associated signalling pathways were to a high degree influenced after acute radiation both *in vitro* and *in vivo*. Moreover, a regulatory network involving miRNA and GTPases is indicated in this process.

## Discussion

The aim of this study was to elucidate the biological mechanisms involved in the acute radiation-induced side-effects on learning and memory as seen in patients treated with radiotherapy. We first used immortalised primary neurons from mouse hippocampus (HT22 cells) to get information about affected signalling pathways. We then validated these data *in vivo* by using the lowest radiation dose inducing non-transient signalling pathway alterations in the cell culture system to irradiate male NMRI mice on postnatal day ten with subsequent analysis of the affected signalling pathways in the hippocampus and cortex.

Pathway analysis of the *in vitro* data showed that the doses of 1.0 Gy and 4.0 Gy, but not 0.5 Gy, significantly altered the expression of proteins functionally involved in RhoGDI signalling, actin cytoskeleton signalling, regulation of actin-based motility by Rho as well as signalling by Rho family GTPases.

Rho family GTPases are key regulators of actin cytoskeleton and are essential for orchestrating spine and synapse morphology [37]. Their activity is controlled at least in part via RhoGDI proteins [38]. Overall, these pathways shared several proteins such as Rac1, PAK, LIMK and cofilin that all are constituents of the Rac1-Cofilin pathway. Quantification of Rac1 and miR-132 levels demonstrated a dose- and time-dependent reduction in both only at doses of 1.0 Gy and 4.0 Gy and 24 hours post irradiation. miR-132 is known to indirectly positively regulate Rac1 activity by blocking the GTPase-activating protein p250GAP [39], [40]. Thus a decrease in miR-132 would lead to a decrease in Rac1 activity as we observed.

Similar signalling pathways were affected in murine hippocampus and cortex at 1.0 Gy 24 hours after the exposure as in the cell culture system. Also the Rac1 and miR-132 levels were similarly down-regulated as *in vitro* at this experimental set-up. Overall, the decrease in Rac1 expression and activity as observed *in vitro* and *in vivo* may lead to a presumptive aberrant actin remodelling in dendritic spines.

It has been shown that selective deletion of Rac1 in excitatory neurons *in vivo* affects spine structure, impairs synaptic plasticity and spatial learning [41]. Chemical inactivation of Rac1 impairs long-term plasticity in the mouse hippocampus [32]. In contrast, activation of the cerebral Rac1 leads to rearrangement of cerebral actin cytoskeleton and improvement of learning and memory for several weeks in mice [42]. Similarly, it was demonstrated that inhibition of Rac1 leads to disruption of F-actin flow in hippocampal rat neuron cultures [43] and to a progressive elimination of dendritic spines in rats [44].

Recently, a miRNA profiling cohort study with Alzheimer's patients illustrated a strong decrease in miR-132 levels in the prefrontal cortex and hippocampus [45]. The deregulation of miR-132 seemed to occur predominately in neurons displaying Tau hyper-phosphorylation [45] emphasising the role of miR-132 in cognitive diseases. Additionally, it was shown that miR-132 is down-regulated in temporal cortical areas and in the CA1 region of hippocampal neurons of human Alzheimer's brain [46].

To get further insight into the downstream effects of Rac1 in the Rac1-Cofilin pathway, we quantified alterations in the end product of this pathway. Cofilin and phospho-cofilin levels were both increased in the hippocampus but not in the cortex after 1.0 Gy exposure (24 hours post-irradiation). It has been shown that also fascin plays an important role in the organisation of actin filament bundles whereas cofilin may play a cooperative role in the disassembly of filopodial actin filaments *in vitro* [47]. Proteomics data of the irradiated hippocampus showed an increase in fascin1 (Fscn1) levels whereas in the cortex we did not observe any significant alterations (Table S2 in File S2). Thus, it remains speculative whether an increase in cofilin and fascin protein levels is necessary for the acute radiation-induced actin remodelling only in the hippocampus but not in the cortex. Dephosphorylated cofilin binds to actin resulting in enhanced filament severing [48] and thus actin depolymerisation. In contrast, phosphorylated cofilin enhances actin filament turnover [49]. However, while phosphorylated cofilin is impaired in actin severing function, dephosphorylated cofilin is not necessarily active, since it may undergo inactivation by other means, including cellular sequestration [50], [51]. Thus, it is important to note that cofilin regulation in the dendritic spine context is probably not a simple switched on- (dephosphorylated cofilin) and switched off- (phosphorylated cofilin) mechanism but is also dependent on the relative concentration of cofilin to actin [51], [52].

We suggest that radiation exposure alters the basal expression of cofilin and leads thus to aberrant actin signalling and processing in dendritic spines. Although we observe that both total cofilin and phospho-cofilin are increased in irradiated hippocampus potentially to equilibrate cofilin/phospho-cofilin ratio in a normal range and allow the recovery from synaptic damages, the relative concentration of actin to cofilin, whether phosphorylated or not, may be consequently changed. This hypothesis has to be confirmed by further experiments such as the evaluation of morphometric parameters relevant to actin/cofilin regulation after irradiation. Nevertheless, imbalances in the actin/cofilin ratio may lead to altered spine morphology. Dendritic spines and their ability to form synapses play an important role in modulating and storing of information [53]. Filamentous actin represents the major cytoskeletal component in dendritic spines to ensure morphological integrity [54], [55]. Thus, it seems likely that morphological defects in spine shape, size and number are dependent on local actin dynamics and signalling. In fact, spines are able to induce rapid actin-based remodelling processes to change their morphology within seconds [56] to react efficiently to stressors such as ionising radiation.

However, it remains enigmatic if the acute alterations in spine architecture signalling pathways we observed here lead to persistent spine morphology changes. Chakraborti et al. showed that a high-dose (10 Gy) irradiation of the brain in young adult mice resulted in alterations in dendritic spine density and morphology in the hippocampus lasting up to one month [57]. Moreover, even doses as used in our study (1.0 Gy) are able to trigger persisting changes in dendritic complexity, synaptic protein levels, spine density and morphology in murine hippocampal neurons [21]. It has also been shown that immature filopodia in the hippocampus were more sensitive to irradiation compared to mature spines [21]. As filopodia are cytoplasmic projections containing actin filaments cross-linked into bundles by Rho family GTPases [43], these data are in good agreement with the acute alterations in the Rac1-Cofilin pathway found in our study. Importantly, the Rac1 protein regulates the synaptic maturation and integration of adult born neurons in the hippocampus [58], [59]. Deregulation of Rac1 may even play an important role in defects of hippocampal adult neurogenesis that are observed in several radiation exposure studies [17]–[20].

Overall, we show that ionising radiation leads to acute changes in cytoskeletal signalling pathways associated with spine morphology *in vitro* and *in vivo*, by altering the molecular players within the Rac1-Cofilin-pathway. An understanding of the signalling pathway alterations after acute radiation exposure is essential in order to prevent immediate side-effects affecting learning and memory in accidentally exposed persons and patients treated with radiotherapy against brain tumours.

## Supporting Information

File S1 **Supporting figures.** Figure S1, Mass spectrometry-based proteomics changes in HT22 cells as a function of time. Venn diagrams of all and shared deregulated proteins as a function of time (4 hours vs. 24 hours) from HT22 cells exposed to 0.5 Gy (A), 1.0 Gy (B) and 4.0 Gy (C) from global proteomics approach; n = 4 for each radiation dose. Detailed protein information (protein name, fold-changes, protein variability) of time-dependent overlapping deregulated proteins is given in D, E and F. Figure S2, Visualisation of cytoskeletal synaptic-plasticity signalling pathways and their overlapping proteins. Visualisation of pathways involved in RhoGDI signalling, regulation of actin-based motility by Rho, regulation by Rho Family GTPases and actin cytoskeleton signalling and their overlapping proteins as highlighted in red boxes. The images were downloaded from Ingenuity Signalling Pathway Analysis (IPA) software. Figure S3, Mass spectrometry-based proteomics of *in vivo* – comparison of brain regions. Overlapping deregulated proteins in hippocampus [H] and cortex [C] with protein names, fold-changes, and protein variability 24 hours post-irradiation using global proteomics approach; hippocampus: n = 4 and cortex: n = 5. Figure S4, Mass spectrometry-based proteomics of *in vivo and in vitro* – comparison of overlapping proteins in a similar experimental set-up. Overlapping deregulated proteins in HT22 cells, hippocampus [H] and cortex [C] at 1.0 Gy 24 hours post-irradiation are shown (A). The protein Gmps that was found deregulated in all analysis is shown with protein name, fold-changes, and protein variability using global proteomics approach (B); HT22 cells and hippocampus: n = 4 and cortex: n = 5. Figure S5, Visualisation of the fold changes of snoRNA135 and ΔCt values between miRNA-135 and snoRNA135 in control and irradiated cells and tissues. The columns represent the fold changes of snoRNA135 in miRNA normalisation (A, C, E) and ΔCt values of the differences between the Ct values of miRNA-135 and snoRNA135 (B, D, and F) with standard errors of the mean (SEM) *in vitro* (HT22 cells: n = 4 for 4 hours and 24 hours post-irradiation) and *in vivo* (hippocampus [H]: n = 3 for 24 hours post-irradiation; cortex [C]: n = 3 for 24 hours post-irradiation) experimental set-ups.
(PDF)

File S2 **Supporting tables.** Table S1, Deregulated proteins found in mass spectrometry-based proteomics in *in vitro* irradiated HT22 cells. Complete detailed list of deregulated proteins (name, unique peptides, n-fold-change, variability and counts per biological replicate) obtained from HT22 cell experiment 4 hours and 24 hours post-irradiation at doses of 0.5 Gy, 1.0 Gy and 4.0 Gy from global proteomics analysis. Deregulated proteins at 1.0 Gy 24 hours after radiation exposure were categorised into protein classes using PANTHER classification system software and the general annotation from UniProt as indicated by an asterisk. Table S2, Deregulated proteins found in mass spectrometry-based proteomics of the hippocampus and cortex of irradiated NMRI mice. Complete detailed list of deregulated proteins in hippocampus and cortex (name, unique peptides, n-fold-change, variability and counts per biological replicate) obtained from NMRI mice experiment 24 hours post-irradiation at doses of 1.0 Gy from global proteomics analysis. Deregulated proteins at 1.0 Gy 24 hours after radiation exposure were categorised into protein classes using PANTHER classification system software and the general annotation from UniProt as indicated by an asterisk.
(XLS)
