# Supplementary material for: Ionising Radiation Immediately Impairs Synaptic Plasticity-Associated Cytoskeletal Signalling Pathways in HT22 Cells and in Mouse Brain: An In Vitro/In Vivo Comparison Study
Source: PLoS One. 2014 Oct 20;9(10):e110464. doi: 10.1371/journal.pone.0110464 (PMC4203799; doi:10.1371/journal.pone.0110464)
Supplement: File S1 — Supporting figures. Figure S1, Mass spectrometry-based proteomics changes in HT22 cells as a function of time. Venn diagrams of all and shared deregulated proteins as a function of time (4 hours vs. 24 hours) from HT22 cells exposed to 0.5 Gy (A), 1.0 Gy (B) and 4.0 Gy (C) from global proteomics approach; n = 4 for each radiation dose. Detailed protein information (protein name, fold-changes, protein variability) of time-dependent overlapping deregulated proteins is given in D, E and F. Figure S2, Visualisation of cytoskeletal synaptic-plasticity signalling pathways and their overlapping proteins. Visualisation of pathways involved in RhoGDI signalling, regulation of actin-based motility by Rho, regulation by Rho Family GTPases and actin cytoskeleton signalling and their overlapping proteins as highlighted in red boxes. The images were downloaded from Ingenuity Signalling Pathway Analysis (IPA) software. Figure S3, Mass spectrometry-based proteomics of in vivo – comparison of brain regions. Overlapping deregulated proteins in hippocampus [H] and cortex [C] with protein names, fold-changes, and protein variability 24 hours post-irradiation using global proteomics approach; hippocampus: n = 4 and cortex: n = 5. Figure S4, Mass spectrometry-based proteomics of in vivo and in vitro – comparison of overlapping proteins in a similar experimental set-up. Overlapping deregulated proteins in HT22 cells, hippocampus [H] and cortex [C] at 1.0 Gy 24 hours post-irradiation are shown (A). The protein Gmps that was found deregulated in all analysis is shown with protein name, fold-changes, and protein variability using global proteomics approach (B); HT22 cells and hippocampus: n = 4 and cortex: n = 5. Figure S5, Visualisation of the fold changes of snoRNA135 and ΔCt values between miRNA-135 and snoRNA135 in control and irradiated cells and tissues. The columns represent the fold changes of snoRNA135 in miRNA normalisation (A, C, E) and ΔCt values of the differences between the Ct [file pone.0110464.s001.pdf]

SI Figure S1

A

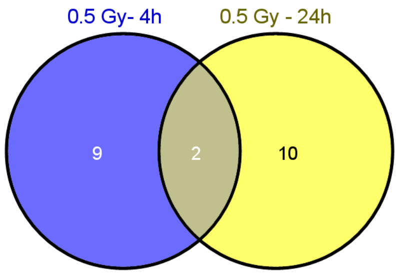

B

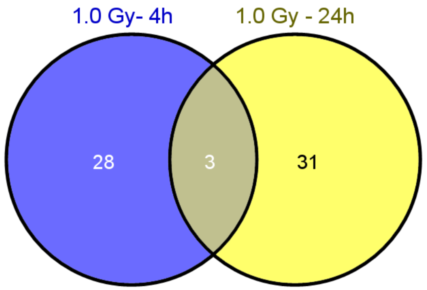

C

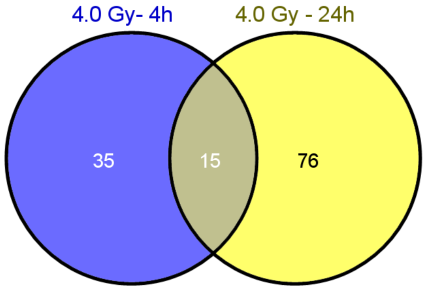

D

Accession number	Symbol	Name	0.5 Gy - 4h	Variability [%]	0.5 Gy - 24h	Variability [%]
ENSMUSP00000029405	Gmps	guanine monophosphate synthetase	1.65	14.9	1.49	13.5
ENSMUSP00000048425	Pebp1	phosphatidylethanolamine binding protein 1	1.36	27.8	1.35	28.3

E

Accession number	Symbol	Name	1.0 Gy - 4h	Variability [%]	1.0 Gy - 24h	Variability [%]
ENSMUSP00000016703	H3f3b	H3 histone, family 3B	3.63	10.2	2.16	27.5
ENSMUSP00000029405	Gmps	guanine monophosphate synthetase	2.51	20.4	1.73	13.5
ENSMUSP00000023235	Eef1d	eukaryotic translation elongation factor 1 delta	2.10	26.7	1.90	23.4

F

Accession number	Symbol	Name	4.0 Gy - 4h	Variability [%]	4.0 Gy - 24h	Variability [%]
ENSMUSP00000130479	Gspt1	G1 to S phase transition 1	1.65	13.4	1.43	12.5
ENSMUSP00000128967	Immt	inner membrane protein, mitochondrial	1.52	28.1	1.31	20.6
ENSMUSP00000126790	Mcm4	minichromosome maintenance deficient 4 homolog	1.40	27.0	1.39	6.9
ENSMUSP00000103365	Rpl18	ribosomal protein L18	-1.31	9.6	-1.32	8.3
ENSMUSP00000008812	Rps18	ribosomal protein S18	-1.32	8.4	-1.34	2.0
ENSMUSP00000000756	Rpl13	ribosomal protein L13	-1.33	11.9	-1.37	7.6
ENSMUSP00000100998	Rps15	ribosomal protein S15	-1.34	16.4	-1.44	1.1
ENSMUSP00000016463	Slc25a5	solute carrier family 25 (mitochondrial carrier, adenine nucleotide translocator), member 5	-1.37	8.3	-1.47	22.3
ENSMUSP00000079180	Rps25	ribosomal protein S25	-1.40	22.8	-1.41	2.2
ENSMUSP00000079356	Hist1h1b	histone cluster 1, H1b	-1.40	3.7	-1.60	3.8
ENSMUSP00000063714	Arhgdia	Rho GDP dissociation inhibitor (GDI) alpha	-1.51	6.7	-1.39	6.6
ENSMUSP00000114135	Rpl24	ribosomal protein L24	-1.56	12.0	-1.52	8.9
ENSMUSP00000027127	Sf3b1	splicing factor 3b, subunit	-1.59	26.5	-1.31	22.1
ENSMUSP00000131489	Rpl14	ribosomal protein L14	-1.60	18.3	-1.52	3.4
ENSMUSP00000031617	Rpl6	ribosomal protein L6	-1.65	26.1	-1.41	3.3

SI Figure S2

RhoGDI signalling

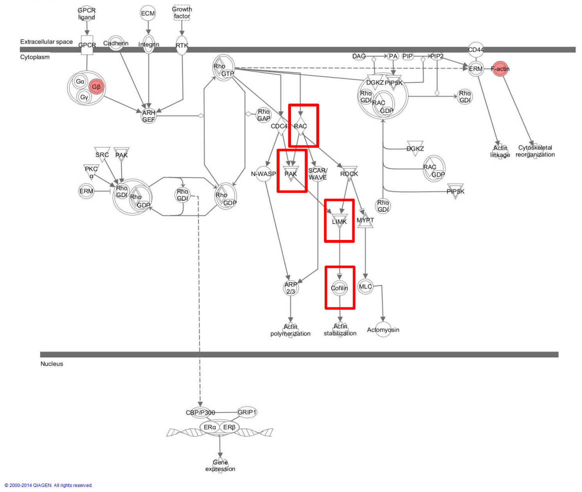

Regulation of Actin-based Motility by Rho

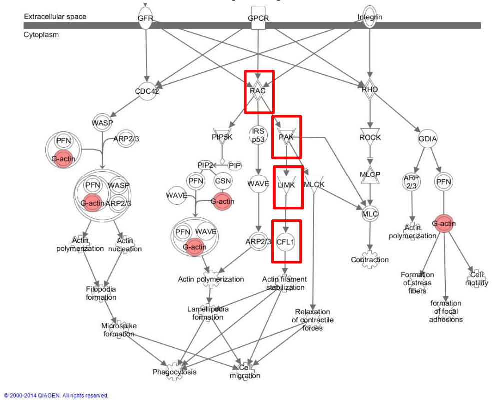

Regulation by Rho Family GTPases

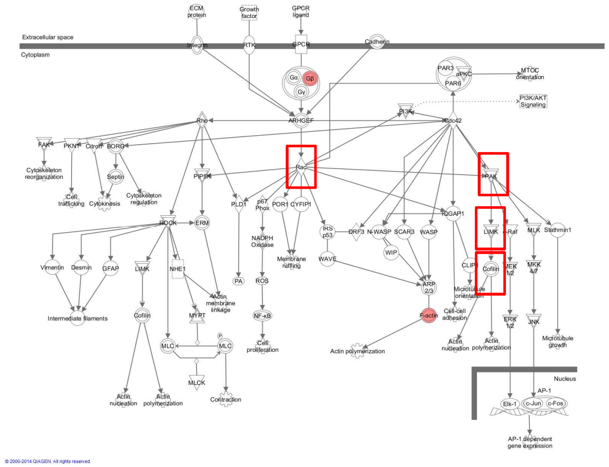

Actin Cytoskeleton Signalling

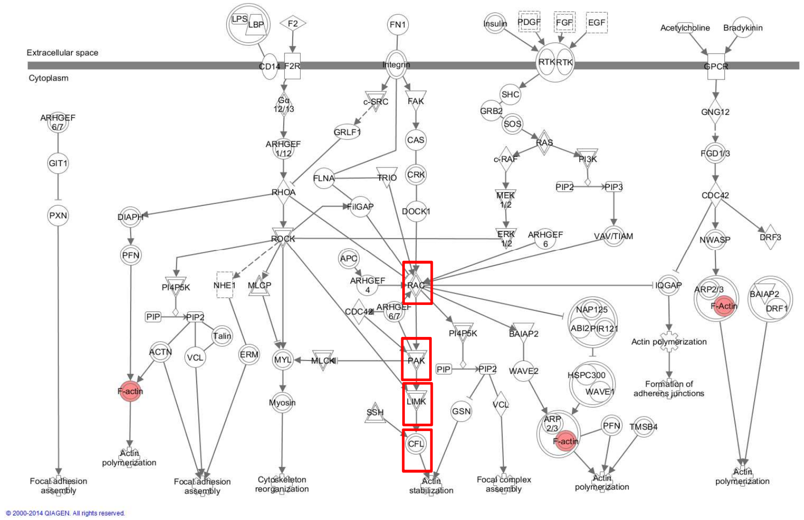

Signalling pathway	Overlapping proteins
RhoGDI Signalling	Rac, PAK, LIMK, Cofilin
Actin Cytoskeleton Signalling	
Regulation of Actin-based Motility by Rho	
Signalling by Rho Family GTPases	

SI Figure S3

Accession number	Symbol	Name	1.0 Gy - Hippocampus	Variability [%]	1.0 Gy - Cortex	Variability [%]
ENSMUSP00000007130	Ctnnb1	catenin (cadherin associated protein), beta 1	3.18	24.9	1.65	19.7
ENSMUSP00000111516	Cdh2	cadherin 2	3.06	29.1	2.15	23.8
ENSMUSP00000066304	Nlgn3	neuroligin 3	2.68	28.9	1.98	27.3
ENSMUSP00000126883	Ank2	ankyrin 2, brain	2.67	25.9	1.77	8.4
ENSMUSP00000026743	Uqcrc1	ubiquinol-cytochrome c reductase core protein 1	2.47	19.1	2.12	18.5
ENSMUSP00000029405	Gmps	guanine monophosphate synthetase	1.96	22.9	1.77	26.1
ENSMUSP00000092658	Rab7	RAB7, member RAS oncogene family	1.48	10.6	1.41	13.9
ENSMUSP00000030940	Gnb1	guanine nucleotide binding protein (G protein), beta 1	1.39	2.5	1.32	27.1

SI Figure S4

A

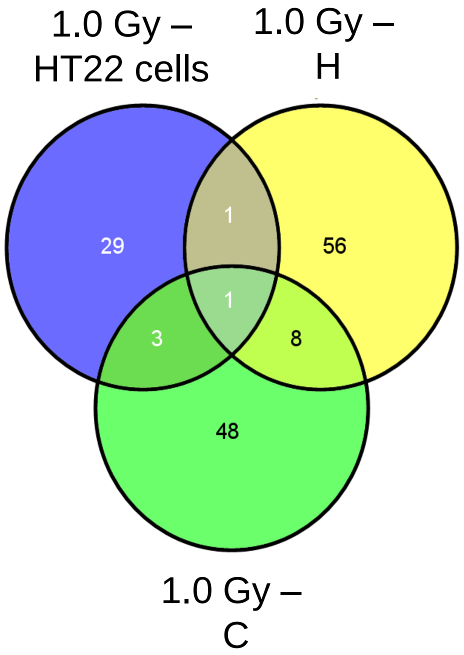

B

Symbol	Name	1.0 Gy - Hippocampus	Variability [%]	1.0 Gy - Cortex	Variability [%]	1.0 Gy - HT22 cells	Variability [%]
Gmps	guanine monophosphate synthetase	1.96	22.9	1.77	26.1	1.73	13.5

SI Figure S5

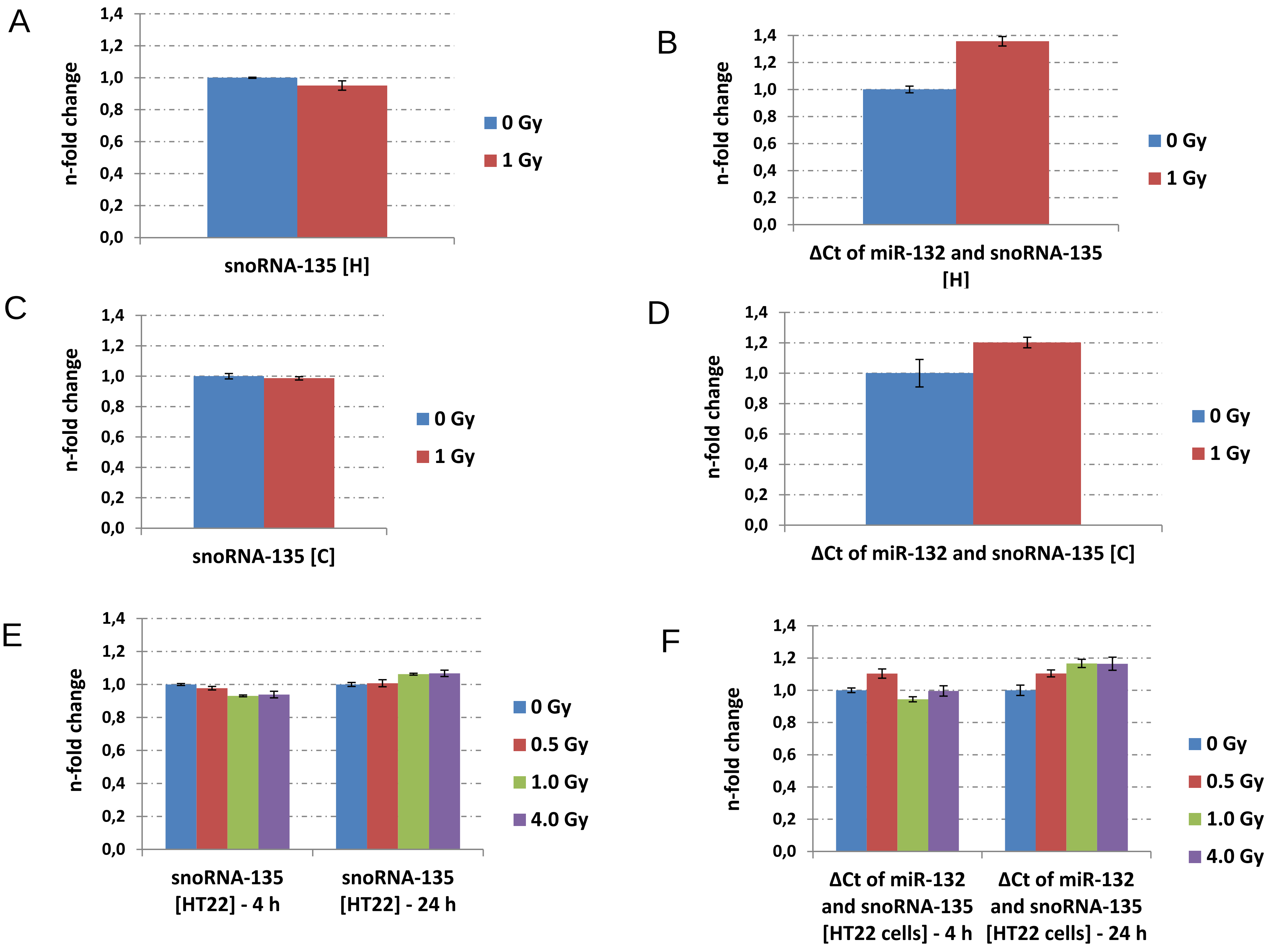

A
n-fold change
1,4
1,2
1,0
0,8
0,6
0,4
0,2
0,0
snoRNA-135 [H]
0 Gy
1 Gy
B
ΔCt of miR-132 and snoRNA-135 [H]
C
snoRNA-135 [C]
D
ΔCt of miR-132 and snoRNA-135 [C]
E
snoRNA-135 [HT22] - 4 h
snoRNA-135 [HT22] - 24 h
0.5 Gy
1.0 Gy
4.0 Gy
F
ΔCt of miR-132 and snoRNA-135 [HT22 cells] - 4 h
ΔCt of miR-132 and snoRNA-135 [HT22 cells] - 24 h
